# Process–Structure Relationships Governing Dimensional Accuracy in Material-Extrusion-Printed PLA-Based Composites

**DOI:** 10.3390/polym18070818

**Published:** 2026-03-27

**Authors:** Alexandra Ana Medruț, Emanoil Linul

**Affiliations:** Department of Mechanics and Strength of Materials, Politehnica University of Timisoara, 1 Mihai Viteazu Blvd., 300222 Timisoara, Romania; alexandra.medrut@student.upt.ro

**Keywords:** material extrusion AM, PLA-based composites, dimensional accuracy, porosity, relative density, extrusion stability, structure-process-dimension relationships

## Abstract

Material extrusion (MEX) additive manufacturing can produce material-dependent variations in dimensional fidelity, internal structure, and deposition stability, even under identical processing conditions. In this study, a comprehensive experimental investigation is conducted on MEX-printed specimens manufactured from a broad set of PLA-based composite materials to quantify these variations and assess their mutual interdependence. Dimensional behavior, internal structural characteristics, and process behavior were systematically investigated using complementary geometric, physical, and deposition-related descriptors. All properties were determined from replicated specimens to ensure statistical robustness, and the resulting datasets were examined using both conventional metrics and multivariate 3D correlation approaches. Compact PLA-based formulations exhibit consistent internal packing, characterized by relative density (RD) values of approximately 0.40–0.46, porosity (ϕ) levels around 55–60%, reduced (≤0.15%) density variability (CV), and small (−0.4–0.0%) volumetric deviations (ΔV). These features reflect stable extrusion and predictable dimensional response. In contrast, foamed, fiber-reinforced, and organic-filled composites display reduced internal packing (RD < 0.40), increased ϕ (>60%), elevated CV (0.27–0.58%), and systematically larger positive ΔV (up to +1.4%), indicating a higher sensitivity to process-induced heterogeneity. Multivariate correlations further reveal that volumetric dimensional distortion is jointly governed by internal packing efficiency and extrusion stability. Overall, the results demonstrate that dimensional accuracy in MEX of PLA-based composites arises from coupled structure–process interactions rather than isolated material or process parameters. The experimental framework proposed here provides quantitative guidance for material selection and process optimization aimed at enhancing geometric fidelity in composite filament fabrication.

## 1. Introduction

Additive manufacturing (AM) has transformed polymer-based fabrication by enabling the direct production of complex geometries with reduced tooling requirements and high design flexibility [1,2,3]. Among the various AM technologies, material extrusion (MEX) has become one of the most widely adopted approaches in both academic research and industrial practice due to its accessibility, process adaptability, and compatibility with a broad range of thermoplastic materials [4,5,6,7]. The technology is extensively employed for rapid prototyping, functional components, and experimental studies, with polylactic acid (PLA) remaining the most prevalent polymer owing to its low processing temperature, relatively low thermal shrinkage, and stable extrusion behavior compared to other commodity thermoplastics [8,9,10]. Despite these advantages, achieving reliable dimensional accuracy (DA) remains a critical limitation of MEX, constraining its broader use in applications requiring tight geometric tolerances and high repeatability [11,12,13]. Dimensional deviations in MEX-fabricated parts originate from the intrinsic characteristics of layer-by-layer deposition, where molten material undergoes complex thermal, rheological, and solidification processes [14,15]. Non-uniform cooling, material shrinkage, interlayer bonding effects, and deposition-induced anisotropy collectively contribute to geometric inaccuracies that accumulate throughout the build [16,17]. Consequently, DA in MEX cannot be interpreted solely as a function of machine resolution or nominal parameter settings, but rather as the outcome of coupled interactions between printing process parameters (PPPs), material composition, and internal structural development [18,19].

A substantial body of the literature has investigated DA in MEX by focusing on the role of fundamental PPPs. Early systematic studies demonstrated that dimensional deviations are direction-dependent, with in-plane dimensions typically exhibiting larger errors than the build direction. Sood et al. [20] showed that shrinkage dominates the deposition plane, while thickness can exhibit systematic oversizing depending on layer stacking behavior, highlighting the anisotropic nature of dimensional errors. Similar directional sensitivity was reported by Hyndhavi et al. [21] and Zisopol et al. [22], who emphasized that optimal parameter combinations differ depending on the dimension considered. Layer thickness (LT) has consistently emerged as one of the most influential PPPs governing DA. Stojković et al. [23] and Bolat and Ergene [24] demonstrated that reduced LT generally improves dimensional stability by enhancing interlayer bonding and reducing thermal gradients, although the magnitude of this effect is material-dependent. Raster angle (RA) and build orientation (BO) further modulate dimensional response by altering bead alignment and cooling behavior. Hyndhavi et al. [21] and Tunçel [25] showed that specific RAs can minimize deviations in selected axes, while Górski et al. [26] reported that BO introduces nonlinear dimensional behavior that cannot be captured by simple relationships. Printing speed (PS) and extrusion temperature (ET) also play a critical role. Ardeljan et al. [27] demonstrated the existence of an optimal PS at which dimensional deviations are minimized while maintaining productivity, whereas Frunzaverde et al. [28,29] showed that elevated ETs increase dimensional deviations due to enhanced melt flow and delayed solidification. Collectively, these studies confirm that DA in MEX arises from multivariate interactions among PPPs rather than isolated effects.

Beyond PPPs, material composition has been identified as a decisive factor influencing DA. Comparative studies on unfilled polymers indicate that materials with lower thermal expansion coefficients and more stable melt behavior tend to exhibit superior dimensional fidelity. Bolat and Ergene [24] and Li et al. [30] showed that PLA generally achieves higher DA than PETG or ABS under comparable conditions, a trend also confirmed by Hyndhavi et al. [21] and Alsoufi et al. [31]. The introduction of fillers and reinforcements significantly alters the dimensional response. Fiber-reinforced and metal-filled composites exhibit distinct extrusion behavior and internal architecture, often increasing anisotropy or inducing systematic oversizing. Nassar et al. [32] demonstrated that carbon fiber reinforcement modifies dimensional stability during post-processing, while Tian et al. [33] and Darsin et al. [34] showed that composite formulations containing rigid or metallic fillers require careful PPP control to limit geometric deviations. Organic and foamed fillers introduce additional complexity: Farzadi et al. [35] and Baraheni et al. [36] reported that porous or irregular fillers promote structural openness and increased dimensional variability due to disrupted consolidation. Material additives such as pigments and functional modifiers further influence dimensional behavior. Hanon et al. [37], Cojocaru et al. [38], and Frunzaverde et al. [28,29] demonstrated that filament color and additive chemistry affect thermal absorption and melt viscosity, leading to measurable differences in DA even under identical processing conditions. These findings collectively indicate that DA in MEX is intrinsically material-dependent and closely linked to formulation and internal structure development.

Recent studies have emphasized that geometric deviations alone are insufficient to fully characterize dimensional performance. Internal structural descriptors such as bulk density (ρ_bulk_), porosity (ϕ), and packing efficiency (RD, relative density) provide critical insight into the mechanisms governing dimensional stability. Baraheni et al. [36] showed that ρ_bulk_ is dominated by infill-related architecture and correlates with DA, while Boschetto et al. [39] demonstrated that internal filament geometry strongly influences geometric deviations. Mass-related indicators and deposition efficiency metrics have also been proposed as complementary descriptors. Hanon et al. [37] and Solouki et al. [40] highlighted that discrepancies between theoretical and measured mass, as well as manufacturing time (MT), reflect process stability and repeatability. Such indicators provide indirect yet valuable information on extrusion consistency and consolidation behavior, linking deposition dynamics to final dimensional outcomes.

Given the multivariable nature of DA, numerous optimization methodologies have been proposed. Taguchi-based approaches, gray relational analysis, ANOVA-driven regression models, and multi-objective optimization frameworks have been widely applied to identify PPP combinations that minimize dimensional deviations while balancing competing objectives. Sood et al. [20], Sharma et al. [41], and Fountas et al. [42] demonstrated that such methods can effectively improve DA and productivity simultaneously. However, a common limitation across these studies is the implicit treatment of structure, process stability, and dimensional response as largely independent outputs. Although multi-criteria optimization integrates multiple performance metrics, the underlying physical relationships linking internal structure formation, extrusion stability, and DA are often not explicitly quantified. As a result, predictive understanding of why certain materials exhibit superior dimensional performance under identical processing conditions remains limited.

Despite the extensive adoption of PLA-based composite filaments in MEX, establishing transferable relationships between material formulation and dimensional accuracy remains challenging. Prior studies often evaluate dimensional deviations under material-specific parameter tuning or tailored process windows, which can mask formulation-driven effects and complicate cross-material comparison. In addition, commercial filament compositions are frequently reported only at a generic level (e.g., “wood-filled” or “metal-filled”), with limited provenance and incomplete quantitative descriptors of matrix grade and additive/filler content. Finally, dimensional accuracy is commonly treated as an isolated outcome, while the coupled roles of deposition stability (e.g., flow consistency) and internal structural formation (e.g., bulk density/porosity) are less systematically integrated into a single comparative framework. These limitations restrict mechanistic interpretation and reduce the practical usefulness of reported trends for process control and material selection.

To address these gaps, the present study provides a unified, process-controlled comparison of fifteen commercially available PLA-based filaments spanning the dominant formulation strategies encountered in MEX (unfilled PLA; mineral-, organic/bio-, and fiber-reinforced composites; fine functional-additive grades; a foaming system; and metallic-effect pigment formulations). All materials were printed under identical processing conditions to isolate formulation effects. We introduce a consistent classification and nomenclature scheme used throughout the manuscript and compile datasheet-derived provenance and material descriptors (Supplementary Table S1) to improve traceability. Using this approach, we quantify and connect (i) dimensional accuracy, (ii) internal structural characteristics, and (iii) deposition stability metrics, enabling interpretation of how different filler strategies influence the coupled process–structure–accuracy response of MEX-printed PLA-based parts.

## 2. Materials and Methods

### 2.1. Materials

PLA was selected as the base polymer for this study due to its extensive use in material extrusion additive manufacturing, favorable processing window, and well-documented suitability as a matrix for composite filaments [43,44]. Owing to its relatively stable melt behavior and compatibility with a wide range of fillers, PLA enables systematic investigation of how different reinforcement strategies affect extrusion behavior, internal structural formation, and DA under identical processing conditions [45,46].

A total of fifteen PLA-based materials were investigated, including unfilled PLA as well as PLA-based grades incorporating mineral fillers, organic/bio-fillers, fibrous reinforcements, fine functional additives, a dedicated foaming system, and appearance-grade metallic-effect formulations. To ensure clarity and consistency throughout the manuscript, each material is identified using a unique abbreviation. In addition to the complete material designations, Table 1 summarizes the corresponding functional roles, material categories, and sustainability aspects of the investigated filaments. This table provides the reference nomenclature and classification framework used consistently across all subsequent figures, analyses, and discussions.

The investigated filament set was selected to enable a representative, process-controlled comparison across the dominant PLA-based formulation strategies commonly adopted in commercial MEX filaments, rather than to cover all possible fillers. The selection was guided by (i) market relevance and availability of widely used commercial grades, (ii) inclusion of distinct filler morphologies associated with different expected process–structure mechanisms during extrusion (dense particulate powders, rigid mineral particulates, short fibers, porous organic/bio-fillers, fine functional additives, and a dedicated foaming system), and (iii) controlled comparability by restricting the matrix family to PLA-based materials and applying identical processing conditions for all prints. This approach enables formulation-dependent effects on deposition stability, internal structural formation, and dimensional accuracy to be compared and interpreted consistently across material classes. Accordingly, the material set is intended to span the principal commercial formulation archetypes encountered in PLA-based MEX filaments, enabling cross-class comparison under controlled PPPs using traceable, datasheet-derived descriptors (Table S1) rather than polymer-grade variables that are not uniformly disclosed for commercial products.

**Figure 1:**
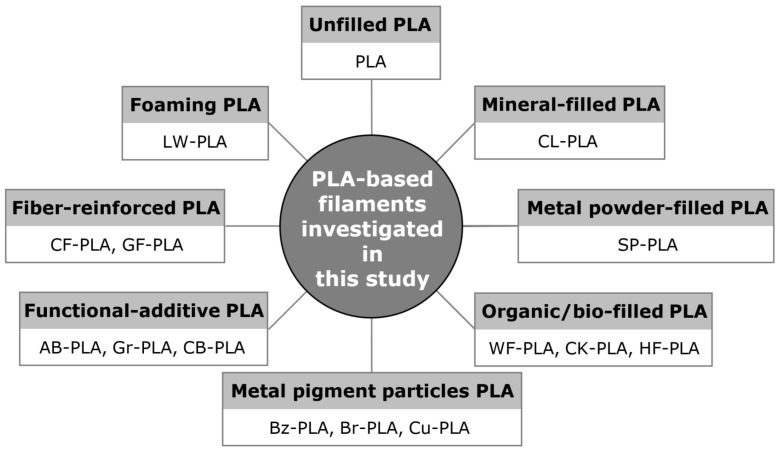
Classification of the investigated PLA-based materials by filler type.

Based on filler type and composite formulation, the investigated materials were grouped into eight distinct categories, as schematically illustrated in Figure 1. This classification reflects the dominant structural role of the filler within the PLA matrix and provides a consistent framework for interpreting the experimental results.

*Unfilled* PLA was used as the reference material, representing a dense polymer without fillers and providing the baseline for assessing material-dependent structural and dimensional deviations. *Metal powder-filled* PLA composites contain dense metallic powders (e.g., steel), which increase the deposited mass and may affect local compaction and thermal behavior during extrusion, while not inherently generating open porosity. *Metal pigment particles* PLA grades form a separate class, in which metallic appearance is achieved primarily through pigments or effect additives (e.g., brass/bronze/copper look), without the high-load metal powder formulation typical of metal powder-filled composites; these formulations may still influence flow and surface quality. *Organic-filled* PLA composites incorporate natural fillers such as wood fibers or cork. Their irregular morphology and intrinsic porosity tend to promote structural openness and higher variability in internal packing, with possible effects on extrusion continuity and dimensional stability. *Mineral-filled* PLA composites include rigid inorganic particles (stone/mineral type), introducing a stiff particulate phase that can modify bead consolidation and packing without inducing active expansion. *Fiber-reinforced* PLA composites contain short fibers of carbon or glass; their anisotropic character may influence melt flow, inter-road bonding, and directional dimensional response. *Organic/bio-filled recycled* PLA (rPLA) composites (hemp-related variant) represent a distinct organic/bio-filled recycled subset within the organic-filled class, where the manufacturer describes hemp-associated organic bio-fillers/by-products; the exact filler content and morphology are not disclosed, but the formulation can still affect packing variability and dimensional stability. PLA with fine *functional additives* involves low fractions of dispersed agents such as silver nanoparticles, graphene, or carbon black, primarily to impart antibacterial or electrical properties; these do not dominate pore structure but can still affect flow and uniformity. Finally, *foaming* PLA forms a distinct class, in which an expansion mechanism activated during extrusion causes volumetric growth, density reduction, and pronounced pore formation.

All materials were supplied as filaments with a nominal diameter of 1.75 mm and were processed under identical printing conditions. This approach ensures that the observed differences in structural, dimensional, and process-related behavior can be attributed primarily to material formulation rather than to variations in machine settings or PPPs.

### 2.2. Specimen Fabrication

All specimens were manufactured using a MEX process under strictly controlled and identical PPPs in order to isolate material-dependent effects from process-induced variability. The printed geometry consisted of cubic specimens with a nominal edge length of 30 mm, selected to enable accurate evaluation of dimensional fidelity, internal structure, and volumetric behavior. The 3D model was designed using CATIA V5R21 CAD software (Dassault Systèmes, Vélizy-Villacoublay, France) and subsequently processed using Ultimaker Cura 5.0.0 (UltiMaker B.V., Utrecht, The Netherlands) slicing software, compatible with the Original Prusa i3 MK3S MEX printer (Prusa Research a.s., Prague, Czech Republic).

A nozzle diameter of 0.4 mm was employed in combination with a fixed LT of 0.2 mm, ensuring a balance between geometric resolution and deposition stability across all materials. Each specimen was printed with three outer layers, providing sufficient shell thickness to minimize surface-related artifacts while preserving sensitivity to internal packing effects. The ET was set to 220 °C for all materials, while the build platform was maintained at 60 °C to promote adequate first-layer adhesion and reduce thermal gradients during printing. A constant PS of 20 mm/s was selected to favor stable extrusion and consistent bead formation, particularly for composite filaments containing fillers with diverse morphologies. Internal material distribution was controlled through a uniform infill density of 30%, using a star-shaped infill pattern, which was chosen for its ability to promote isotropic load distribution and consistent internal connectivity [13,19,46,47]. The flow rate was uniformly adjusted to 0.95 in order to compensate for minor over-extrusion tendencies and to improve DA across the full material set.

**Figure 2:**
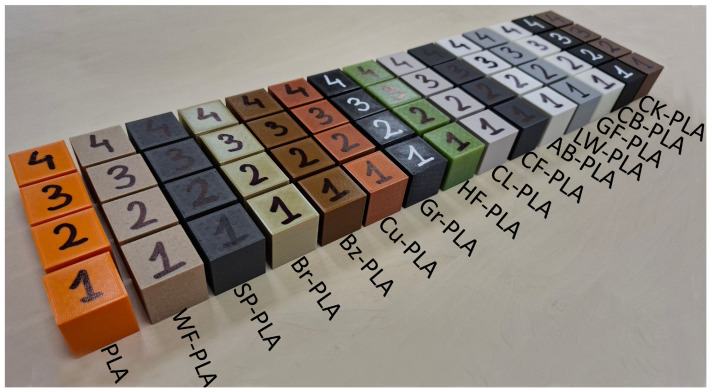
Overview of the fabricated PLA-based specimens produced via MEX.

All primary PPPs are summarized in Table 2, while all remaining slicer settings were kept constant throughout the study. Representative printed specimens are illustrated in Figure 2, providing a visual overview of the MEX-fabricated sample set.

### 2.3. Measurement and Data Processing

#### 2.3.1. Specimen Geometry and Experimental Measurements

**Figure 3:**
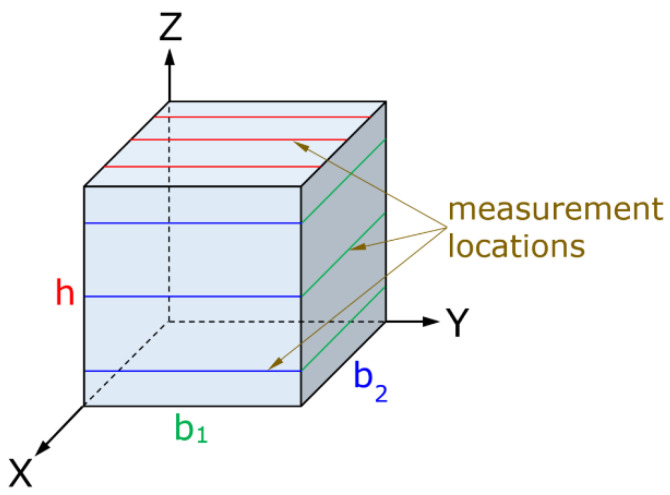
Schematic illustration of the cubic specimen showing the build axes (X, Y, and Z), measured dimensions (h, b_1_, and b_2_), and measurement locations.

For each specimen, the following three orthogonal dimensions were measured: the build direction height (h) and two in-plane dimensions (b_1_ and b_2_), defined as mutually orthogonal directions within the XY plane. A representative schematic of the measured cube, indicating the orientation of the measured axes, is provided in Figure 3 to clarify the adopted dimensional reference system. All measurements were conducted after completion of the printing process, without post-processing or conditioning.

The printing orientation was identical for all specimens, with the model reference aligned at X = 0, Y = 0, and Z = 0 in the build coordinate system. Linear dimensions were measured using a digital, model HG07675A (OWIM GmbH & Co. KG, Neckarsulm, Germany), with a resolution and accuracy of ±0.01 mm. To improve measurement reliability and account for potential local geometric deviations, each dimension was measured at three distinct locations along the corresponding axis (at both extremities and at the midpoint), and the arithmetic mean of these three readings was taken as the representative value for that axis. This procedure was applied consistently to all specimens.

For each material type, four replicated specimens were evaluated, enabling both average dimensional trends and specimen-to-specimen variability to be quantified. The nominal reference value used for dimensional deviation analysis was 30 mm for all axes, corresponding to the designed cube geometry.

Specimen mass was measured using an analytical balance, model PCB 350-3 (KERN & SOHN GmbH, Balingen, Germany) with a readability of ±0.001 g. The MT was kept constant for all specimens, regardless of material formulation or reinforcement type, with a fixed build duration of 76.75 min per specimen. All measured values were retained in the subsequent analysis to preserve the full experimental variability.

#### 2.3.2. Definition of Derived Properties and Data Processing

All dimensional, structural, and process-related descriptors were derived from the primary measurements according to consistent analytical definitions. Calculations were first performed at the specimen level and subsequently aggregated at the material level by computing mean values and standard deviations (SDs) across the four replicated specimens. No data filtering or outlier removal was applied at any stage of the analysis [30,48,49,50,51,52,53].

Derived properties (e.g., RD, porosity, volumetric deviation, and deposition-stability indicators) were computed from the primary measurements following a consistent post-processing procedure. For transparency and reproducibility, the full set of definitions, equations, and processing steps is provided in the Supporting Information (Supplementary Section S2).

## 3. Results and Discussions

This section presents a comprehensive evaluation of DA, internal structure, and process-related behavior of MEX-printed PLA-based composites. The results are analyzed to clarify how material composition and filler morphology govern extrusion stability, internal packing, and anisotropic dimensional response. By integrating geometric, physical, and deposition-related descriptors, the analysis reveals the coupled structure–process mechanisms that control final part quality.

Because the investigated filaments are commercial grades with non-uniform disclosure of polymer-level descriptors, the discussion relates inter-material differences to established PLA composite material-family effects primarily through the experimentally observed process–structure signatures (e.g., RD/ϕ/CV and MFII/VBSI), rather than through molecular-weight or stereochemical parameters. Accordingly, matrix-grade effects cannot be fully decoupled from filler effects for each commercial product.

### 3.1. Geometrical Properties

The dimensional characterization of the printed specimens provides a direct and quantitative assessment of geometric fidelity and repeatability across the investigated PLA-based materials. Because all samples were printed as nominal 30 × 30 × 30 mm^3^ cubes, deviations in the three measured axes (h, b_1_ and b_2_), can be interpreted in terms of both systematic bias (mean shift from nominal) and process stability (scatter across replicated specimens).

**Figure 4:**
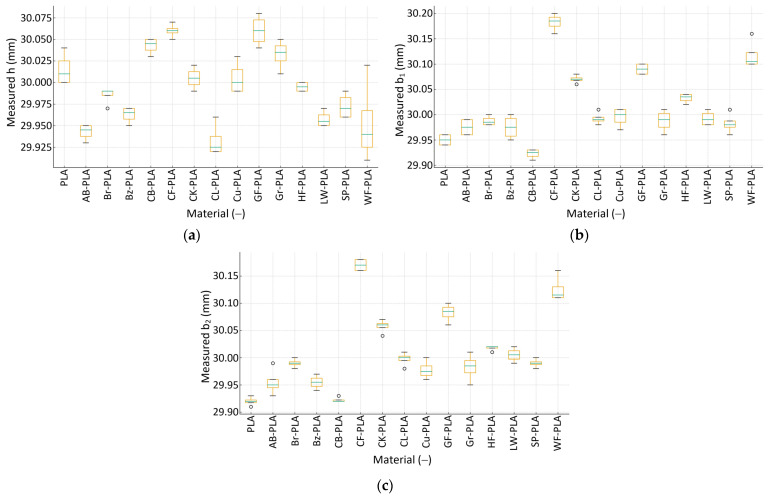
Dimensional variability of the MEX-printed specimens across the three measured axes: (**a**) measured h; (**b**) measured b_1_; (**c**) measured b_2_. The green horizontal line within each box indicates the median value.

Figure 4 illustrates the dimensional variability across all materials in millimeters, highlighting both the mean position relative to the nominal target and the material-specific spread. Overall, the measured dimensions remain close to the 30 mm target, yet clear material-dependent trends are evident. In the height direction h, the mean values span from 29.9325 mm (CL-PLA) to 30.0600 mm (CF-PLA), corresponding to mean offsets of approximately −0.0675 mm to +0.0600 mm from nominal. The SD along h is generally low, with many materials exhibiting about 0.006–0.020 mm, indicating stable layer stacking and consistent Z-axis control. A notable exception is WF-PLA, which shows the largest height scatter (~0.0479 mm), suggesting increased build-to-build variability likely driven by material heterogeneity and less uniform deposition. This elevated h-dispersion is consistent with local under-/over-deposition events that affect layer stacking (e.g., transient flow fluctuations and non-uniform bead flattening), which propagate more directly into Z build variability across replicas.

Greater variability is observed in the lateral dimensions *b_1_* and *b_2_*, which are more sensitive to bead spreading, raster-induced anisotropy, and in-plane thermal contraction. For *b_1_*, mean values range from 29.9225 mm (CB-PLA) up to 30.1825 mm (CF-PLA), i.e., a systematic SD from −0.0775 mm to +0.1825 mm. For b_2_, mean values range from 29.9200 mm (PLA) to 30.1700 mm (CF-PLA), i.e., approximately −0.0800 mm to +0.1700 mm. These results demonstrate that lateral accuracy is more strongly material-dependent than height accuracy. This behavior is expected given the different sensitivity of the in-plane and build-direction dimensions to deposition physics under fixed machine settings. In the XY plane, the final geometry is governed by effective bead width and lateral spreading, which are directly influenced by formulation-dependent melt rheology, filler-driven flow behavior, and the quality of inter-road fusion and consolidation, while in-plane thermal contraction further amplifies small deviations. In contrast, the height direction is largely constrained by the imposed LT and the discrete Z-axis positioning, such that material-driven changes in bead shape translate less directly into systematic height errors when PPPs are held constant. In terms of dispersion, WF-PLA shows the highest spread in b_1_ (0.0287 mm), while AB-PLA and Gr-PLA show among the higher spreads in b_2_ (0.0252 mm for AB-PLA and 0.0250 mm for Gr-PLA). Such increases in in-plane scatter are consistent with more complex flow behavior and heterogeneous packing during deposition. Importantly, the most pronounced systematic oversizing in both in-plane directions is observed for CF-PLA (b_1_ = 30.1825 mm, b_2_ = 30.1700 mm), whereas several compact materials exhibit mild undersizing (e.g., PLA in b_2_ at 29.9200 mm), indicating that filler morphology can shift the balance between shrinkage and expansion/poor compaction.

**Figure 5:**
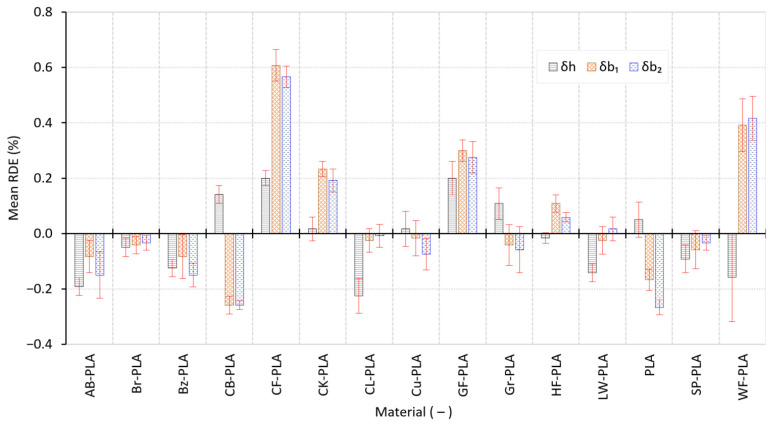
Mean RDEs and associated variability across the three measured axes.

While Figure 4 captures dispersion and absolute dimensional positioning in mm, Figure 5 quantifies DA using the relative dimensional error. The RDE confirms the anisotropic nature of error propagation. In height (h), the mean RDE values across materials span approximately 0.0167% (HF-PLA) up to 0.2250% (CL-PLA), with SDs reaching up to 0.0995% for the most variable cases. In contrast, the in-plane axes show substantially larger errors for certain composites: in b_1_, the mean RDE ranges from 0.0417% (Br-PLA) up to 0.6083% (CF-PLA), and in b_2_ from 0.0250% (CL-PLA) up to 0.5667% (CF-PLA). The elevated in-plane RDE values for CF-PLA are consistent with the oversizing observed in Figure 4 (positive shifts of +0.1825 mm in b_1_ and +0.1700 mm in b_2_), indicating that the fiber-filled formulation introduces systematic lateral expansion and/or reduced consolidation. Conversely, compact PLA-based formulations generally maintain low RDE with limited scatter, reflecting stable melt flow and predictable thermal contraction under the fixed processing conditions.

**Figure 6:**
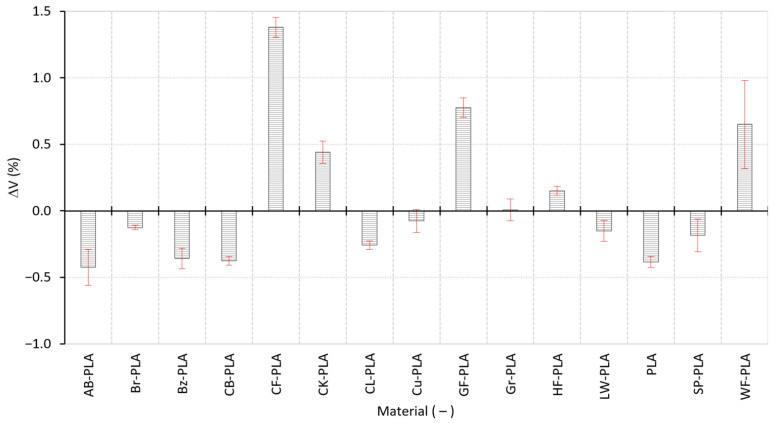
ΔVs of the MEX-printed specimens relative to the nominal volume.

To integrate axis-specific deviations into a single volumetric indicator, Figure 6 reports the volumetric dimensional deviation ΔV relative to the nominal cube volume. This global metric confirms that small lateral biases, even when height remains stable, can accumulate into significant volumetric distortion. Across materials, the mean ΔV spans from a minimum of approximately −0.4244% (AB-PLA) to a maximum of +1.3808% (CF-PLA), indicating a broad material-driven spread in volumetric fidelity. Many compact materials cluster around small negative ΔV values (predictable shrinkage), while highly heterogeneous composites show positive ΔV (volumetric oversizing). The reproducibility of the volumetric response is also strongly material-dependent: the largest ΔV scatter is observed for WF-PLA (0.3314%), consistent with its elevated dimensional scatter in Figure 4 (notably along h and b_1_). The pronounced ΔV scatter of WF-PLA is consistent with the behavior of wood-filled composites, where irregular and partially porous bio-filler particles can increase melt heterogeneity and promote less uniform bead formation and consolidation. Such effects typically manifest as local deposition fluctuations and spatially variable packing, which elevate replica-to-replica dispersion in both dimensions and internal structure, and therefore propagate into higher ΔV variability under identical PPPs. This interpretation is also aligned with the increased dimensional scatter previously observed for WF-PLA (notably along h and b1), indicating a higher sensitivity to deposition-induced heterogeneity compared with more compact particulate- or fiber-filled grades. By contrast, more stable formulations exhibit smaller ΔV SDs, indicating repeatable dimensional outcomes.

**Figure 7:**
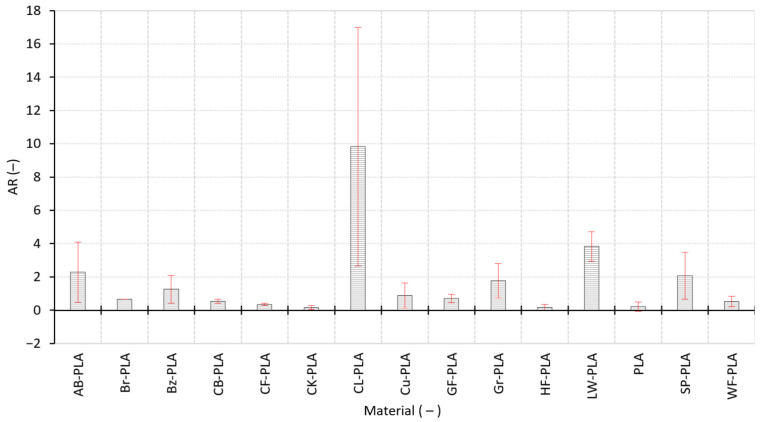
Anisotropic dimensional response of the MEX-printed specimens expressed by the AR.

Figure 7 complements the axis-wise dimensional evaluation by quantifying the anisotropic in-plane response through the anisotropy ratio. Low AR values indicate nearly isotropic behavior between the two lateral directions, whereas elevated AR values reflect a strong directional sensitivity of dimensional response, typically associated with raster-dependent consolidation, nonuniform cooling, and filler-driven flow instabilities. Most compact materials exhibit low anisotropy, with mean AR below 1.0, including PLA, CK-PLA, HF-PLA, CF-PLA, WF-PLA, CB-PLA, Br-PLA, and GF-PLA. This indicates that, for these materials, lateral dimensional deviations are comparatively balanced between b_1_ and b_2_, consistent with stable bead geometry and predictable thermal contraction under fixed PPPs.

In contrast, several formulations show pronounced anisotropy and/or high replica-to-replica variability, highlighting sensitivity to deposition direction and local packing heterogeneity. The most extreme case is CL-PLA, with mean AR of 9.833 ± 7.167, indicating not only strong anisotropy but also substantial variability among replicas, which is consistent with unstable in-plane consolidation and large scatter in lateral response. Such extreme AR values can occur when one lateral direction accumulates a systematic bias (e.g., direction-dependent bead spreading/compaction) while the orthogonal direction remains near-neutral, so the ratio amplifies small absolute asymmetries into a large AR. Elevated anisotropy is also observed for LW-PLA, AB-PLA, SP-PLA, and Gr-PLA. These results suggest that, beyond global shrinkage/oversizing trends captured by RDE and ΔV, certain materials exhibit a distinctly direction-dependent dimensional behavior, likely driven by filler morphology (foaming structure in LW-PLA, metallic particles in SP-PLA, and additive/filler-induced rheological shifts in AB-PLA and Gr-PLA) and by the interaction between extrusion stability and RA deposition. Therefore, Figure 7 confirms that dimensional fidelity in the XY plane is not only material-dependent but also directionally biased for specific composites, reinforcing the need to interpret b_1_ and b_2_ jointly rather than assuming isotropic in-plane contraction.

Overall, the dimensional evaluation reveals a clear material-dependent transition from predictable and repeatable geometry (compact PLA-based materials) toward increased anisotropy, scatter, and volumetric distortion as filler-induced heterogeneity increases. The height direction remains comparatively stable across the material set, whereas the lateral dimensions capture the dominant sensitivity to deposition dynamics, bead spreading, and internal packing variability. These geometric findings establish the basis for the subsequent sections.

### 3.2. Physical and Structural Properties

The analysis of bulk density and porosity provides essential insight into the internal structure of the MEX-printed specimens and represents a direct structural counterpart to the geometric deviations discussed in Section 3.1. By quantifying the effective material packing achieved during deposition, these parameters clarify how filler morphology, melt flow behavior, and consolidation efficiency influence the final internal architecture of PLA-based composites.

**Figure 8:**
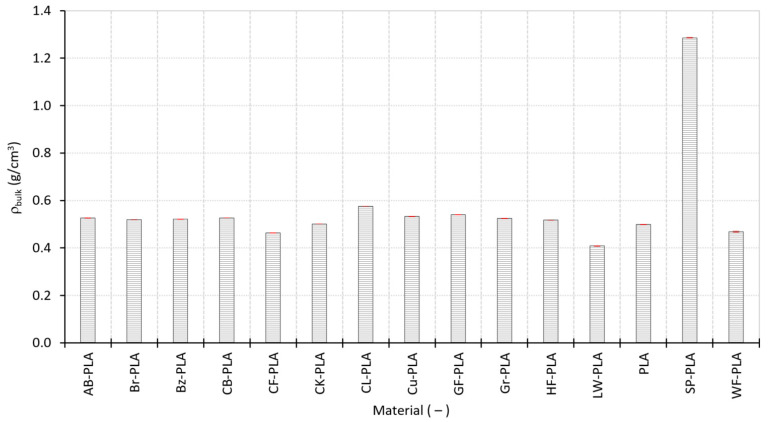
Bulk density of the MEX-printed specimens for all investigated materials.

Figure 8 reports the measured bulk density ρ_bulk_ values together with their SDs. Across most investigated materials, ρ_bulk_ values cluster within a relatively narrow range centered around 0.50–0.55 g/cm^3^, indicating broadly comparable levels of internal packing under the selected processing conditions. For unfilled or mildly modified PLA formulations (such as PLA, AB-PLA, Br-PLA, Bz-PLA, CB-PLA, Gr-PLA, and HF-PLA), the observed densities indicate consistent consolidation and limited void growth. The very small SDs (generally <0.001 g/cm^3^) further confirm that these materials exhibit stable deposition and reproducible internal structure across replicas.

A moderate increase in ρ_bulk_ is observed for CL-PLA (0.576 g/cm^3^) and GF-PLA (0.539 g/cm^3^), suggesting improved compaction or reduced void fraction relative to standard PLA. This behavior may be attributed to altered rheology or enhanced bead spreading, promoting more effective inter-road bonding. Cu-PLA (0.534 g/cm^3^) occupies a similar density range, reflecting the influence of metallic particles on local packing efficiency without inducing extreme heterogeneity.

In contrast, several materials exhibit significantly reduced ρ_bulk_, reflecting increased internal void content and incomplete consolidation. LW-PLA, designed to introduce controlled foaming, shows the lowest ρ_bulk_ at 0.409 g/cm^3^, consistent with volumetric expansion and reduced mass deposition. CF-PLA and WF-PLA also display substantially lower densities, indicating that fibrous fillers disrupt melt continuity and limit effective packing. CK-PLA occupies an intermediate position, suggesting partial consolidation combined with cork-induced void retention. These lower-density materials also show larger SDs, reflecting increased sensitivity to local flow fluctuations and specimen-to-specimen variability.

A distinct outlier is SP-PLA, which exhibits a markedly higher ρ_bulk_ of 1.286 g/cm^3^. This exceptional value reflects the presence of heavy metallic fillers and highlights the fundamental difference between mass-based density and internal packing efficiency. Despite its high ρ_bulk_, SP-PLA does not necessarily imply a void-free structure, but rather a substantially increased mass per unit volume due to filler composition.

**Figure 9:**
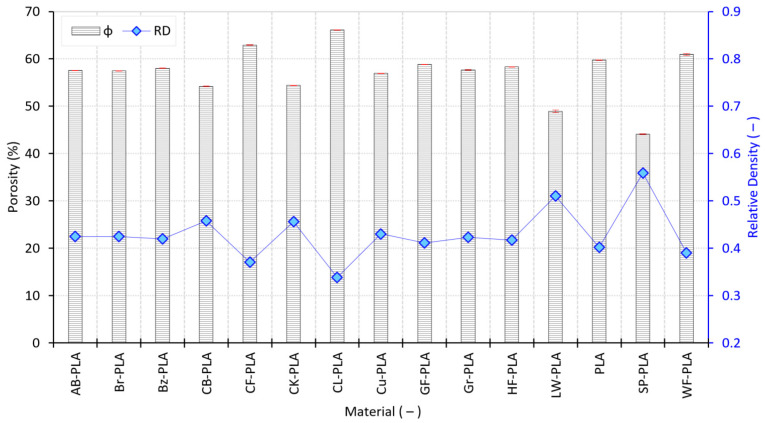
Porosity and relative density variations in the MEX-printed specimens.

The corresponding porosity ϕ values, presented in Figure 9, provide a normalized structural interpretation by relating bulk density (ρ_bulk_) to the nominal material density (ρ_mat_). For most PLA-based formulations, ϕ values are high, typically within the 55–60% range, indicating that MEX printing inherently produces structures with substantial internal void content under the selected PPPs. Materials such as PLA, AB-PLA, Br-PLA, Bz-PLA, CB-PLA, Cu-PLA, GF-PLA, HF-PLA, and Gr-PLA cluster tightly within this ϕ interval, with small SDs that mirror the stability observed in their ρ_bulk_ measurements. CB-PLA (54.20%) and CK-PLA (54.40%) lie slightly below this range, while SP-PLA exhibits the lowest porosity (44.08%). Materials with reduced ρ_bulk_ generally exhibit elevated ϕ, although the magnitude of this effect depends strongly on filler morphology. CL-PLA (66.11%), CF-PLA (62.93%) and WF-PLA (60.95%) show the highest ϕ levels in the dataset, reflecting the combined effects of fiber-induced flow disruption, reduced inter-road consolidation, and increased void connectivity. In contrast, LW-PLA, despite its low ρ_bulk_, exhibits a markedly lower ϕ (48.91%), consistent with its foamed internal structure in which volumetric expansion is accompanied by a reduced effective void fraction relative to fibrous composites. This apparent contrast arises because ϕ is computed relative to the nominal material density: foaming reduces the effective filament/part density through expansion, so a lower bulk does not necessarily translate into a proportionally higher “void fraction” as defined by this normalization. In other words, LW-PLA combines volumetric expansion with a comparatively lower normalized void fraction than fiber/wood-filled grades, where poor inter-road consolidation drives high interconnected void content. CK-PLA displays intermediate behavior, with ϕ values (54.40%) close to the compact PLA-based cluster, consistent with partial void retention associated with cork fillers and nonuniform packing.

Figure 9 also reports the RD, expressed as an adimensional parameter, enabling direct comparison across materials with different nominal densities. Compact PLA-based materials (including PLA, AB-PLA, Br-PLA, Bz-PLA, GF-PLA, HF-PLA, Gr-PLA, Cu-PLA, CB-PLA, and CK-PLA) exhibit RD values spanning from 0.40 to 0.46, confirming a comparable degree of internal compaction despite compositional differences. Materials with more open or heterogeneous internal architectures occupy the lower end of the spectrum, with CL-PLA, CF-PLA, and WF-PLA exhibiting RD < 0.40. Among all investigated materials, CL-PLA exhibits the lowest RD value (0.339), indicating the most reduced bulk-to-nominal density ratio in the dataset. LW-PLA (0.511) represents a distinct case among the investigated materials, reflecting the combined effect of foaming-related volumetric expansion and density normalization, and it is associated with a comparatively reduced porosity level relative to the compact group. In contrast, SP-PLA displays the highest RD value (0.559), reflecting its elevated ρ_bulk_ due to metallic filler loading despite the presence of internal voids. Because RD reflects the ratio between measured bulk density and nominal material density, heavy metallic loading can raise the deposited mass per unit volume and shift RD upward even when voids are still present, i.e., the structure can be “dense-by-mass” while remaining partially porous.

**Figure 10:**
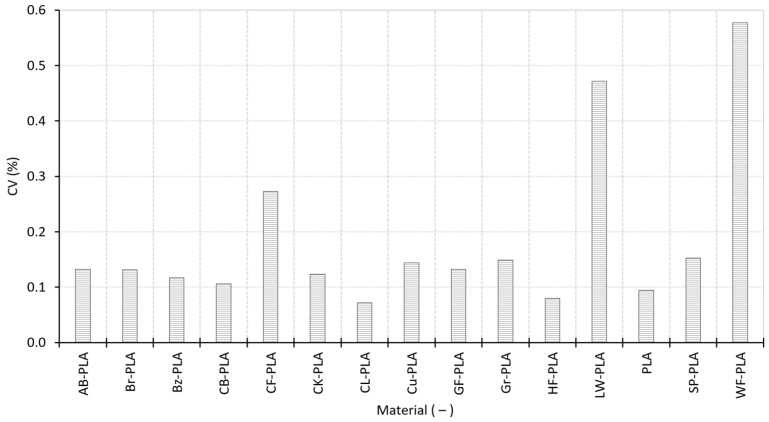
Coefficient of variation in specimen density for all materials.

While ρ_bulk_, ϕ, and RD describe the average compactness of the printed structures, Figure 10 evaluates the repeatability of consolidation by quantifying the coefficient of variation in bulk density CV across replicated specimens. This property captures the specimen-to-specimen dispersion of density and therefore reflects how consistently each formulation reproduces the same internal packing state under identical process settings.

The dataset shows that density repeatability is generally good, but it is clearly material-dependent. The lowest CV is observed for CL-PLA (0.0720%), HF-PLA (0.0799%), and PLA (0.0943%), indicating highly reproducible mass distribution and consolidation for these formulations. Notably, a low CV reflects repeatability of average bulk density across replicas and does not preclude direction-dependent dimensional effects (e.g., anisotropic bead spreading), which can drive AR without necessarily increasing specimen-to-specimen density dispersion. A second, broader group (CB-PLA, Bz-PLA, CK-PLA, Br-PLA, AB-PLA, GF-PLA, Cu-PLA, and Gr-PLA) exhibits slightly higher but still limited CV, spanning roughly 0.10–0.15%. Importantly, the spread within this band remains modest, confirming that (despite compositional differences) the selected PPPs lead to a stable and repeatable average packing state.

A distinct increase in CV appears for the more heterogeneous composites. SP-PLA (0.1527%) remains close to the main cluster, suggesting that metallic loading increases mass content without strongly destabilizing consolidation repeatability. In contrast, CF-PLA (0.2732%) shows a clear step upward, consistent with fiber-induced perturbations that can locally alter flow continuity and inter-road packing. The highest CV is observed for LW-PLA (0.4721%) and WF-PLA (0.5771%), reflecting the strong sensitivity of foamed or wood-filled systems to small fluctuations in extrusion conditions and local packing efficiency. These materials are therefore more prone to replica-to-replica density dispersion, which is consistent with their increased dimensional scatter and ΔVs discussed in Section 3.1.

In general, Figure 10 demonstrates that the process is broadly reproducible, but the repeatability margin narrows or widens depending on filler morphology and the stability of internal structure formation. The systematic escalation from 0.07 to 0.15% (compact PLA-based materials) toward 0.27–0.58% (fiber/foamed composites) provides a quantitative link between material heterogeneity, consolidation consistency, and the downstream dimensional behavior later captured in the multivariate correlations.

Collectively, the combined analysis of ρ_bulk_ (Figure 8), ϕ and RD (Figure 9), and CV (Figure 10) establishes a clear and internally consistent structural stratification among the investigated materials. Compact PLA-based formulations are characterized not only by similar mean density and ϕ levels, but also by low CV, indicating that their internal packing state is both well-defined and reproducible. In contrast, materials incorporating foaming agents, fibrous reinforcements, or porous fillers exhibit a coupled response as follows: reduced ρ_bulk_, increased ϕ, and systematically elevated CV. This combination reveals that structural openness and consolidation instability emerge simultaneously rather than independently. Importantly, the CV results demonstrate that differences in internal structure are primarily material-driven, not stochastic process artifacts. These structural characteristics provide a direct explanation for the dimensional anisotropy and volumetric oversizing identified in Section 3.1 and establish the structural foundation for the process-level indicators examined in Section 3.3. The density-porosity-variability framework developed here therefore acts as a critical bridge linking geometric accuracy to deposition behavior and extrusion stability.

### 3.3. Process-Related Properties and Deposition Efficiency

The process-related characterization provides additional insight into how each PLA-based composite behaves during deposition, complementing the geometric and physical analyses discussed previously. Since all specimens were manufactured under identical printing conditions and identical MT (76.75 min for every specimen), differences in deposition performance and stability can be attributed primarily to material-dependent melt-flow behavior, rheological response, and the way fillers influence extrusion and consolidation. In this context, filler dispersion quality and matrix-filler interfacial compatibility (set by the manufacturer’s compounding route and not fully disclosed for all commercial grades) are recognized as underlying factors that may modulate flow consistency and bead consolidation and thereby contribute to the observed differences in deposition stability and dimensional response. To capture these effects, the following four indicators were evaluated: the mass deposition rate, the build rate efficiency, the mass-flow irregularity index, and the volumetric build stability index. Because filament grades differ in nominal density (ρ_mat_; Table S1), we report both mass-based (R_m_, MFII) and volume-based (VBSI and ΔV) indicators; the latter provide a density-aware basis for comparing deposition stability and its link to DA across materials. Together, these metrics quantify not only how much material is delivered per unit time, but also how efficiently that mass is transformed into built volume and how repeatable the process outcome is across replicated specimens.

**Figure 11:**
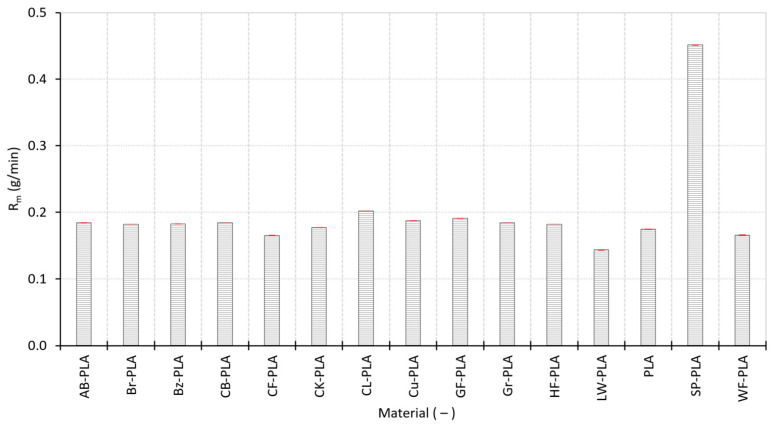
Mass deposition rate of the MEX-printed specimens for all investigated materials.

As illustrated in Figure 11, mass deposition rate R_m_ exhibits clear material dependence despite constant MT. Most materials cluster tightly around 0.175–0.205 g/min, with very small SDs (±0.00015 to ±0.00025 g/min) indicating highly consistent mass delivery from specimen to specimen. The “baseline” PLA response is 0.17477 g/min, while several lightly modified formulations (Br-PLA, Bz-PLA, AB-PLA, and CB-PLA) exhibit closely comparable behavior. This narrow cluster confirms that, for these filaments, the extrusion system maintains a stable throughput under fixed machine settings, consistent with their modest dimensional dispersion in Section 3.1 and their low CV in Section 3.2.

Distinct deviations appear for materials with strongly altered composition or internal structure. LW-PLA records the lowest R_m_ (0.14356 g/min), reflecting reduced delivered mass per unit time consistent with its lightweight/foamed formulation and its low ρ_bulk_ in Section 3.2 (0.409 g/cm^3^). CF-PLA and WF-PLA also fall below the main cluster, aligning with their heterogeneous consolidation behavior (high ϕ and RD < 0.40 in Section 3.2) and their elevated geometric distortions (ΔV up to +1.3808% for CF-PLA in Section 3.1). At the opposite extreme, SP-PLA exhibits a markedly higher R_m_ (0.45162 g/min), more than twice any other formulation. This result is consistent with its exceptionally high ρ_bulk_ (1.286 g/cm^3^) and high RD (0.559), indicating a high mass flux associated with metal-filled filament extrusion.

**Figure 12:**
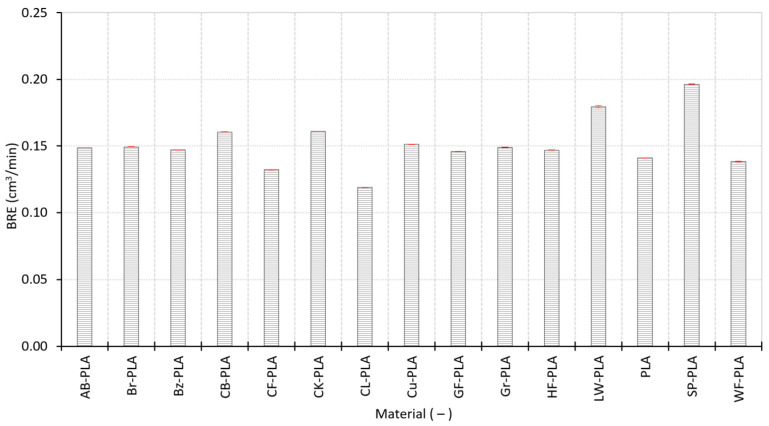
Build rate efficiency of the MEX-printed specimens, normalized to the material density.

Build rate efficiency BRE normalizes deposition behavior to provide a density-compensated indicator of volumetric productivity. The BRE values highlight contrasts that are not visible from R_m_ alone. In the intermediate group, many materials (including PLA, Br-PLA, Bz-PLA, AB-PLA, HF-PLA, and CB-PLA) cluster around BRE of 0.14–0.16 cm^3^/min (Figure 12), consistent with their comparable internal packing states and stable geometric response. CB-PLA (0.1605 cm^3^/min) and CK-PLA (0.1611 cm^3^/min) lie at the upper edge of this cluster (~0.16 cm^3^/min). Clear deviations appear for materials with extreme structural or compositional effects as follows: LW-PLA reaches 0.179 cm^3^/min, reflecting high volumetric build effectiveness relative to mass deposited. The highest BRE is observed for SP-PLA (0.196 cm^3^/min) in this dataset, indicating that the combined effect of its high mass throughput and density normalization yields a very high volumetric productivity index under the present definition of BRE. In contrast, the lowest BRE values are observed for CL-PLA (0.119 cm^3^/min), CF-PLA (0.132 cm^3^/min), and WF-PLA (0.138 cm^3^/min), consistent with their reduced consolidation efficiency (high ϕ and/or lower RD) and increased geometric deviation. This behavior for SP-PLA is consistent with its comparatively low porosity (ϕ ≈ 44.08%) and high compaction (RD ≈ 0.559), i.e., a large amount of built volume produced per time in a structurally dense state.

**Figure 13:**
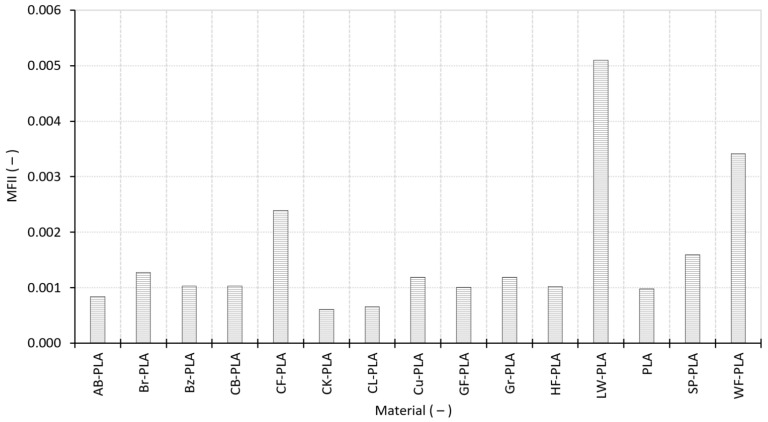
Mass flow irregularity index describing extrudate stability across replicated specimens.

Mass-flow irregularity index MFII describes the stability of mass delivery across replicated specimens and is computed as the coefficient of variation in R_m_. According to Figure 13, most materials exhibit very low MFII, confirming stable extrusion under fixed PPPs. The lowest MFII values are observed for CK-PLA (0.000609) and CL-PLA (0.000664), followed by AB-PLA and PLA, indicating minimal specimen-to-specimen variability in mass throughput. A moderate cluster spans approximately 0.0010–0.0013, including Bz-PLA, CB-PLA, HF-PLA, and Br-PLA. In contrast, the highest MFII values are recorded for the most heterogeneous materials: LW-PLA, WF-PLA, and CF-PLA. These elevated values indicate intermittent or less repeatable mass delivery, consistent with filler-driven flow disturbance (fibers/wood) and the foaming-related variability of LW-PLA. Importantly, these materials are also the ones that exhibited higher dimensional scatter and larger ΔV deviations in Section 3.1, providing a process-level explanation for their reduced geometric fidelity.

**Figure 14:**
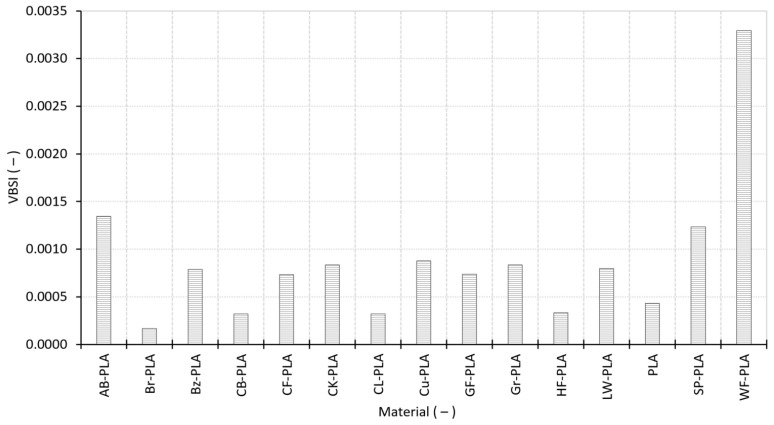
Volumetric build stability index quantifying the repeatability of the constructed volume.

Volumetric build stability index VBSI provides a volumetric counterpart to MFII and is computed as the coefficient of variation in the built volume. Figure 14 shows that most materials exhibit very low VBSI values, confirming that final volume is generally reproducible even when some mass-flow variability exists. This indicates that moderate mass-throughput fluctuations can partially average out over the full build and do not necessarily translate into proportional volume scatter, especially when bead placement is geometrically constrained by the toolpath under fixed slicing parameters. The lowest VBSI is observed for Br-PLA (0.000167), followed by HF-PLA, CB-PLA and CL-PLA, a pattern consistent with their stable dimensional response (Section 3.1) and low CV (Section 3.2). The highest VBSI is recorded for WF-PLA (0.003293), indicating the largest specimen-to-specimen volumetric dispersion; this is fully consistent with WF-PLA also having the highest ΔV SD (0.3314%) in Section 3.1 and the highest CV (0.5771%) in Section 3.2. Elevated volumetric dispersion is also observed for AB-PLA and SP-PLA, while Cu-PLA and Gr-PLA fall in a moderate range.

Overall, the process-related indicators demonstrate that deposition performance is strongly governed by intrinsic material characteristics even under constant printing conditions. Lightweight and structurally heterogeneous materials (LW-PLA, WF-PLA, and CF-PLA) exhibit reduced mass throughput and, more importantly, significantly higher irregularity indices (MFII and VBSI), which aligns with their higher dimensional scatter and ΔVs (Section 3.1) and their more open or heterogeneous internal structures (Section 3.2). Conversely, compact and stable formulations cluster tightly in R_m_ and BRE and maintain low MFII/VBSI, indicating reproducible extrusion and geometric formation. These findings provide the process-level basis for the multivariate correlations presented in Section 3.4, where deposition stability and internal compaction are shown to interact directly with global DA.

### 3.4. Derived Properties and Correlations

A comprehensive understanding of material performance in material-extrusion AM requires examining how geometric, physical, and process-related descriptors interact, rather than treating them independently. In the previous Section 3.1, Section 3.2 and Section 3.3, dimensional fidelity, ρ_bulk_, ϕ and deposition efficiency were analyzed as separate responses. In contrast, the multivariate framework introduced in this section integrates these descriptors into shared parametric spaces, allowing the dominant mechanisms to be identified from their combined evolution. By projecting three key indicators into 3D correlation maps, interaction patterns and material clusters become apparent that are not visible in conventional two-variable plots, providing an integrated view of how filler morphology, internal packing and extrusion behavior jointly control final structural and dimensional outcomes.

**Figure 15:**
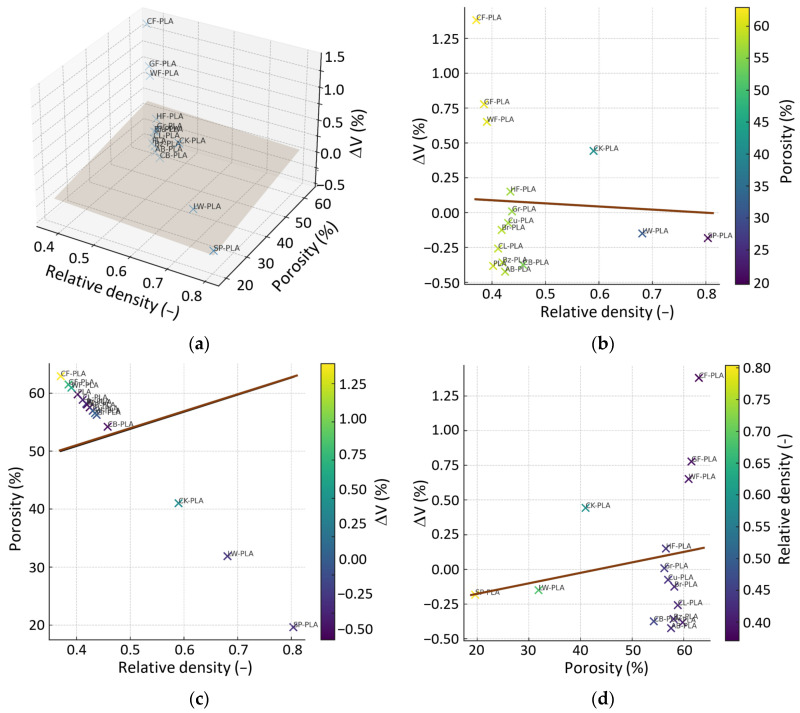
Multivariate relationship among RD, ϕ, and ΔV: (**a**) 3D regression plane; (**b**) ΔV vs. RD, colored by ϕ; (**c**) ϕ vs. RD, colored by ΔV; (**d**) ΔV vs. ϕ, colored by RD. The brown line denotes the regression trend, while the dark shaded area in (**a**) denotes the regression plane.

The first correlation map (Figure 15) links the internal structural descriptors (RD and ϕ) to the global ΔV. A compact and well-defined cluster is observed for materials with RD in the range 0.40–0.46 and ϕ around 55–60%. Within this region, ΔV remains slightly negative, typically between about −0.38% and −0.10%. This group includes PLA, AB-PLA, Br-PLA, Bz-PLA, CB-PLA, CL-PLA, HF-PLA and Gr-PLA, all of which exhibit relatively homogeneous microstructures, limited void connectivity and predictable shrinkage. Their tight grouping around the regression plane indicates that moderate ϕ combined with uniform internal packing leads to stable and reproducible volumetric contraction during cooling, with minimal case-to-case variability.

In contrast, several composites deviate markedly from this structural-dimensional baseline. CF-PLA, GF-PLA and WF-PLA occupy a separate high-distortion region, characterized by substantially higher ΔV values (0.65–1.38%) and lower RD (0.36–0.40), while still presenting comparatively enlarged effective volume. This behavior is consistent with the fact that stiff fibers, irregular particulate fillers and open, interconnected voids disrupt local consolidation and can promote expansion or reduced shrinkage at the macroscopic scale. In practice, reduced consolidation and an altered cooling/shrinkage balance can shift the response from the negative ΔV “shrinkage” regime toward near-zero or positive ΔV, even when the mean packing level (RD) decreases. Their displacement above the regression surface highlights structural openness and heterogeneous packing as strong drivers of positive ΔV.

Intermediate materials populate a transitional zone between the compact PLA cluster and the highly distorted composites. LW-PLA, with its foaming-induced porosity and reduced mass deposition, shows RD significantly below the main PLA group but ΔV values closer to zero, suggesting a partial compensation between expansion due to foaming and reduced compaction forces. CK-PLA, affected by the presence of cork particulates, displays moderate RD and small negative ΔV, indicating nonuniform but overall stable deposition behavior. Taken together, the 3D map and its 2D projections demonstrate that dimensional oversizing is systematically associated with reduced RD and increased structural openness, establishing a clear structural-dimensional linkage in material-extrusion AM.

**Figure 16:**
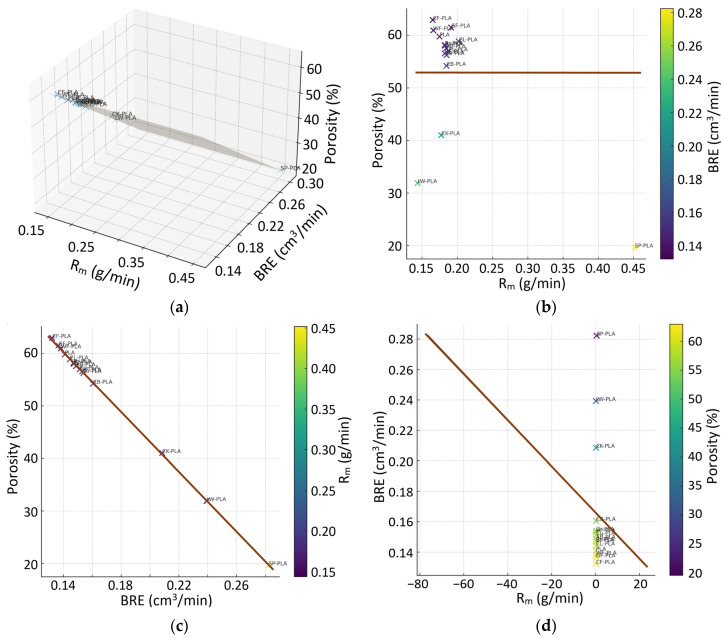
Multivariate relationship among R_m_, BRE, and ϕ: (**a**) 3D regression plane; (**b**) ϕ vs. R_m_, colored by BRE; (**c**) ϕ vs. BRE, colored by R_m_; (**d**) BRE vs. R_m_, colored by ϕ. The brown line denotes the regression trend, while the dark shaded area in (**a**) denotes the regression plane.

The second multivariate map (Figure 16) examines the interplay between deposition behavior (quantified by R_m_ and BRE) and the resulting ϕ. Here, a dominant material cluster is observed for R_m_ values of approximately 0.18–0.22 g/min and BRE values around 0.15–0.18 cm^3^/min, corresponding to porosities near 55–60%. This cluster again includes PLA, AB-PLA, Br-PLA, Bz-PLA, CB-PLA, CL-PLA, HF-PLA and Gr-PLA, and aligns closely with the regression plane within the R_m_-BRE-ϕ space. Their location indicates that a balanced and stable deposition rate leads to consistent internal compaction and moderate, predictable pore content, mirroring the structural stability identified in Figure 15.

SP-PLA appears clearly separated from this PLA-based cluster, occupying the high-throughput, low-ϕ region. It combines the highest R_m_ (~0.45 g/min) and elevated BRE (~0.20 cm^3^/min) with the lowest ϕ in the dataset (~44%). This configuration confirms that the metallic filler content enables high extrusion throughput and enhanced compaction relative to other composites, pushing SP-PLA well below the regression surface toward a dense, low-void state. At the opposite extreme, LW-PLA, driven by its foaming mechanism, exhibits moderate BRE but extremely low density and high ϕ (>30%). Its position far from the main cluster indicates that expansion and void formation dominate over deposition efficiency as the primary factors controlling final pore volume. CK-PLA also separates from the compact PLA region: although its BRE is moderately high, the cork-based filler sustains substantial structural void content, keeping ϕ elevated.

The 2D projections of Figure 16 clarify these trends by isolating pairwise relationships. The R_m_-ϕ and BRE-ϕ maps show a monotonic reduction in ϕ as deposition efficiency increases, while the R_m_-BRE projection highlights distinct groupings dictated by filler morphology and flow behavior. Overall, Figure 16 establishes a robust process–structure linkage: internal compaction in material-extrusion printing emerges from the combined influence of deposition rate and build efficiency, with metal-filled materials achieving the densest structures and highly foamed or organic-filled composites exhibiting the lowest consolidation.

**Figure 17:**
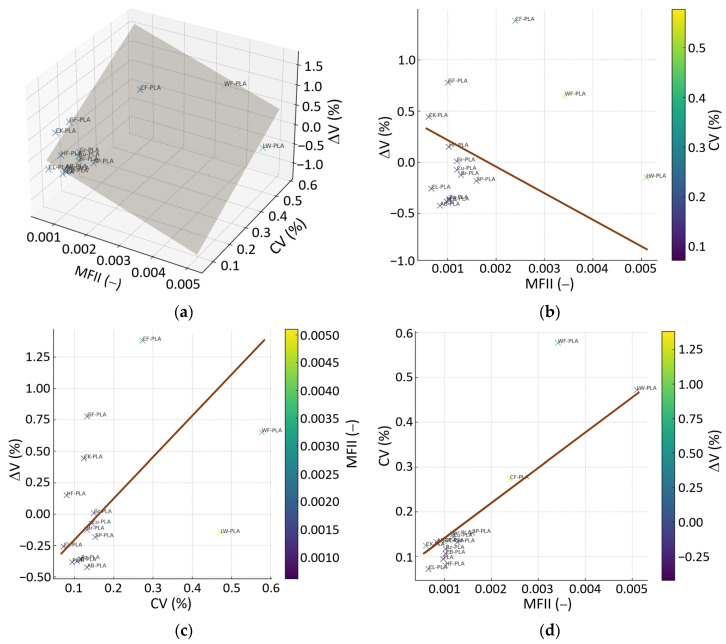
Multivariate relationship among MFII, CV, and ΔV: (**a**) 3D regression plane; (**b**) ΔV vs. MFII, colored by CV; (**c**) ΔV vs. CV, colored by MFII; (**d**) CV vs. MFII, colored by ΔV. The brown line denotes the regression trend, while the dark shaded area in (**a**) denotes the regression plane.

The third multivariate map (Figure 17) integrates extrusion stability (MFII), density uniformity (CV) and volumetric deviation (ΔV), offering a consolidated view of how process-level fluctuations translate into macroscopic dimensional error. The 3D regression plane reveals a clear multivariate dependency, with both MFII and CV contributing jointly and approximately linearly to ΔV. The majority of materials are characterized by low MFII (<0.0012–0.0013) and small CV (<0.15), accompanied by ΔV values close to zero. This group (PLA, AB-PLA, Bz-PLA, CL-PLA, CB-PLA, Br-PLA, HF-PLA and Gr-PLA) clusters tightly around the regression surface, indicating that stable mass flow and uniform internal density distribution result in highly predictable volumetric behavior.

Materials known to exhibit increased structural heterogeneity form a distinct secondary region in this MFII-CV-ΔV space. LW-PLA, WF-PLA and CF-PLA show markedly higher MFII values (0.002–0.005) and elevated CV (0.30–0.60%), together with the largest positive ΔV values in the dataset. Their position relative to the regression plane confirms that extrusion irregularity and density nonuniformity are strong predictors of volumetric inaccuracy: local fluctuations in flow and packing are amplified into global dimensional drift. SP-PLA and CK-PLA occupy an intermediate zone, with MFII and CV values between these two extremes. They reflect consistent extrusion combined with material-specific effects from metal and cork fillers, yielding partially stabilized but still formulation-dependent volumetric responses.

The 2D projections of Figure 17 support this interpretation. The MFII-ΔV map shows a monotonic tendency toward higher distortion with increasing extrusion irregularity, while the CV-ΔV projection confirms that density variability is an equally strong predictor of volumetric expansion or shrinkage. The MFII-CV projection demonstrates that composites with high structural heterogeneity also display less stable deposition, explaining their systematic deviation from the main PLA cluster. Therefore, the combined 3D and 2D analyses underscore that volumetric accuracy in MEX-AM does not arise from any single descriptor but from the coupled effects of flow stability and internal packing uniformity.

The integrated multivariate framework thus reveals a coherent narrative across all structure–process–deformation interactions examined. Materials with compact microstructures, stable melt-flow behavior and balanced deposition kinetics consistently cluster in low-ϕ, low-variability and low-distortion regions across all 3D correlation maps, exhibiting minimal CV, tightly controlled ΔV and reproducible shrinkage patterns. In contrast, composites incorporating fibrous, foamed or highly irregular fillers diverge systematically from this cluster, showing simultaneous increases in ΔV, ϕ, MFII and CV. These coordinated deviations demonstrate that DA in MEX is not governed by a single material or PPP, but by the coupled contributions of structural density, deposition efficiency and extrusion stability. Practically, DA can be manipulated by jointly controlling (i) deposition stability and (ii) internal packing uniformity: first, minimize mass/volume delivery irregularity (low MFII/VBSI) through consistent filament conditioning and calibrated extrusion/feeding, and second, promote repeatable consolidation (higher RD and lower CV) by ensuring adequate thermal conditions and bead overlap that limit underfilling and open void formation. When these two levers are improved simultaneously, the multivariate maps indicate a systematic shift toward the low-distortion region (smaller |ΔV| and reduced scatter), largely independent of the specific filler class. Taken together, the multivariate correlations provide a holistic description of material behavior, clarifying how filler morphology, internal packing and flow regularity interact to shape the final geometric fidelity of printed PLA-based components.

These interpretations should be viewed in the context that the investigated materials are as-purchased commercial filaments, for which polymer-grade variables (e.g., molecular-weight distribution, stereochemical composition, and crystallinity-related characteristics) are not reported consistently across manufacturers; accordingly, the present framework supports robust commercial-grade comparisons based on traceable datasheet descriptors and identical printing conditions, without fully decoupling chemistry- and history-driven effects.

## 4. Conclusions

This study establishes a quantitative understanding of DA in MEX of PLA-based composite materials by linking geometric fidelity to internal structural formation and extrusion stability under identical processing conditions.

- Dimensional behavior is strongly material-dependent. While height deviations remain limited across all materials, in-plane dimensions exhibit systematic variations that lead to anisotropy and volumetric distortion, with ΔVs ranging from predictable shrinkage (−0.4% to 0.0%) to pronounced oversizing in heterogeneous composites (up to +1.4%).
- Internal structure provides the primary explanation for these trends. Compact PLA-based materials exhibit consistent internal packing (RD = 0.40 – 0.46), moderate ϕ (55–60%), and low CV (≤0.15%), resulting in stable and reproducible dimensional outcomes. In contrast, foamed, fiber-reinforced, and organic-filled composites show reduced packing efficiency (RD < 0.40), increased ϕ (>60%), and elevated CV (0.27–0.58%), which directly correspond to larger dimensional distortions.
- Process stability further modulates these effects. Materials with stable mass flow and low extrusion irregularity (MFII = 0.0006 – 0.0012) achieve uniform internal structures and minimal ΔV, whereas higher irregularity (MFII up to 0.005) amplifies CV and dimensional error.

Consequently, mechanistic generalization beyond the commercial-grade level is constrained by the absence of uniform polymer-level characterization across all filaments, and the comparisons are therefore anchored to manufacturer-declared descriptors (Table S1) under identical processing conditions.

Overall, the results demonstrate that DA in MEX of PLA-based composites arises from coupled structure–process interactions rather than isolated parameters. The findings provide practical guidance for material selection and process optimization in applications requiring high geometric fidelity and reproducibility.

## Figures and Tables

**Table 1:** Investigated materials and classification framework used throughout the manuscript.

Abbreviation	Abbreviation Meaning (Generic Name)	Primary Functional Role	Material Category	Sustainability Aspect
PLA	Unfilled PLA (neat PLA)	Baseline reference; process-control benchmark	Unfilled PLA	Bio-based
AB-PLA	Antibacterial PLA	Antibacterial functionality; hygienic surfaces	Functional-additive PLA	Bio-based
Br-PLA	Brass pigment-modified PLA	Melt-flow stability; dimensional response	Metal pigment particles PLA	Hybrid
Bz-PLA	Bronze pigment-modified PLA	Bead consolidation; packing uniformity	Metal pigment particles PLA	Hybrid
CB-PLA	Carbon black-filled PLA	Electrical conductivity; dissipative functionality	Functional-additive PLA	Hybrid
CF-PLA	Carbon fiber-reinforced PLA	Increased stiffness; load-bearing capability	Fiber-reinforced PLA	Hybrid
CK-PLA	Cork-filled PLA	Lightweight behavior; vibration damping	Organic/bio-filled PLA	Bio-based
CL-PLA	Stone-/mineral-filled PLA	Increased density/stiffness; modified bead packing	Mineral-filled PLA	Hybrid
Cu-PLA	Copper pigment-modified PLA	Surface finish uniformity; deposition continuity	Metal pigment particles PLA	Hybrid
GF-PLA	Glass fiber-reinforced PLA	Increased stiffness; thermal resistance	Fiber-reinforced PLA	Hybrid
Gr-PLA	Graphene-modified PLA	Mechanical enhancement; electrical performance	Functional-additive PLA	Hybrid
HF-PLA	Hemp-biofilled recycled PLA (rPLA)	Distinctive texture; packing variability	Organic/bio-filled PLA	Recycled (bio-filled)
LW-PLA	Lightweight (foamed) PLA	Density reduction; in-process light-weighting	Foaming PLA	Hybrid
SP-PLA	Stainless steel powder-filled PLA	High density; thermal mass effects	Metal powder-filled PLA	Hybrid
WF-PLA	Wood-filled PLA	Surface texture; reduced stiffness	Organic/bio-filled PLA	Bio-based

**Table 2:** Main MEX–PPPs used for the fabrication of all PLA-based composite specimens.

Parameter	Value	Unit
Nozzle diameter	0.4	mm
Layer thickness	0.2	mm
Number of outer layers	3	-
Top solid layers	2	-
Bottom solid layers	2	-
Extrusion temperature	220	°C
Bed temperature	60	°C
Positioning accuracy	0.0125	mm
Print speed	20	mm/s
Infill density	30	%
Infill pattern	Stars	-
Printing orientation	X = 0, Y = 0, Z = 0	°
Travel speed	180	mm/s
Flow rate	0.95	-
Top fill pattern	Monotonic	-
Bottom fill pattern	Monotonic	-
Bridges	20	mm/s
Infill/perimeters overlap	25	%
Gap fill	10	mm/s
Fill angle	45	°
Cooling thresholds	15	s

## Data Availability

The original contributions presented in this study are included in the article/Supplementary Materials. Further inquiries can be directed to the corresponding author.

## Supplementary Materials

The following supporting information can be downloaded at https://www.mdpi.com/article/10.3390/polym18070818/s1, Table S1. Datasheet-derived material descriptors and provenance for the investigated filaments. Section S1. Datasheet-derived material descriptors; Section S2. Definition of derived properties and data processing. References [54,55,56,57,58,59,60,61,62,63,64,65,66,67,68] are cited in the supplementary materials.

## Abbreviations, Notations and Symbols

AB-PLA	Antibacterial PLA
ABS	Acrylonitrile butadiene styrene
AM	Additive manufacturing
ANOVA	Analysis of variance
AR	Anisotropy ratio
BO	Built orientation
Br-PLA	Brass pigment-modified PLA
BRE	Built rate efficiency
Bz-PLA	Bronze pigment-modified PLA
CB-PLA	Carbon black-filled PLA
CF-PLA	Carbon fiber reinforced PLA
CK-PLA	Cork-filled PLA
CL-PLA	Stone-/mineral-filled PLA
Cu-PLA	Copper pigment-modified PLA
CV	Coefficient of variation
DA	Dimensional accuracy
ET	Extrusion temperature
GF-PLA	Glass fiber reinforced PLA
Gr-PLA	Graphene-modified PLA
HF-PLA	Hemp-biofilled recycled PLA (rPLA)
LT	Layer thickness
LW-PLA	Lightweight (foamed) PLA
MEX	Material extrusion
MFII	Mass flow irregularity index
MT	Manufacturing time
PETG	Polyethylene terephthalate glycol
PLA	Polylactic acid
PPP	Printing process parameter
PS	Printing speed
RA	Raster angle
RD	Relative density
RDE	Relative dimensional error
rPLA	Recycled PLA
SD	Standard deviation
SP-PLA	Stainless steel powder-filled PLA
VBSI	Volumetric build stability index
WF-PLA	Wood-filled PLA
2D	Two-dimensional
3D	Three-dimensional
b_1,_ b_2_	In-plane specimen widths
h	Specimen height
L_0_	Nominal cube edge length
L_i_	Measured mean dimension along axis i
m_m_	Measured specimen mass
R_m_	Mass deposition rate
t	Print time
V_0_	Nominal/theoretical specimen volume
V_m_	Measured specimen volume
ΔV	Volumetric deviation
δ_i_	Relative dimensional deviation
δ_b1_	Relative dimensional deviation along edge b_1_
δ_b2_	Relative dimensional deviation along edge b_2_
δ_h_	Relative dimensional deviation along build orientation
ρ_bulk_	Bulk/specimen density
ρ_mat_	Nominal material/filament density
σ_p_	Standard deviation of bulk density
σ_m_	Standard deviation of specimen mass
σ_v_	Standard deviation of specimen volume
μ_p_	Mean bulk density
μ_m_	Mean measured specimen mass
μ_v_	Mean specimen volume
ϕ	Porosity
