# Supplementary material for: Process–Structure Relationships Governing Dimensional Accuracy in Material-Extrusion-Printed PLA-Based Composites
Source: Polymers (Basel). 2026 Mar 27;18(7):818. doi: 10.3390/polym18070818 (PMC13075232; doi:10.3390/polym18070818)
Supplement: Supplementary file 1 [file polymers-18-00818-s001.zip › polymers-4104974-supplementary.pdf]

Supplementary Material

S1. Datasheet-derived material descriptors

Table S1. Datasheet-derived material descriptors and provenance for the investigated filaments

Abbreviation	Matrix polymer	Filler / additive	Filler content (wt.%) / size (μm)	Density (g/cm³)	Crystallinity-related characteristics	Melt Flow Rate (MFR) / Melt Flow Index (MFI)	Commercial product name (as purchased)	Manufacturer	Source
PLA	PLA	—	—	1.24	N.R.	N.R.	PLA Original	AzureFilm	[54]
AB-PLA	Modified PLA	Silver nanoparticles (antibacterial)	N.R.	1.24	Tg = 55-60 °C; HDT(B) = 60 °C	N.R.	PLA Antibacterial	Smart Materials 3D (Smartfil)	[55]
Br-PLA	PLA	Dispersed metallic pigment particles	N.R.	1.22	Tg = 57 °C; HDT = 50 °C	MFR = 6 g/10 min (ASTM D1238, 210 °C/2.16 kg)	High Gloss PLA Brass	FormFutura	[56]
Bz-PLA	PLA	Dispersed metallic pigment particles	N.R.	1.24	Tg = 60 °C; HDT = 55 °C	MFR = 6 g/10 min (ASTM D1238, 210 °C/2.16 kg)	ePLA Pearl Bronze	FormFutura	[57]
CB-PLA	PLA	Carbon black	N.R.	1.15	N.R.	N.R.	Electrically Conductive Composite PLA	Proto-pasta	[58]
CF-PLA	PLA	Carbon fiber	N.R.	1.25	N.R.	N.R.	Black PLA-CF	MatterHackers	[59]
CK-PLA	PLA/PHA blend	Cork filler	~30/N.R.	1.1	Tm ≈ 160 °C, Vicat ≈ 46 °C	MFR: n.d. (ISO 1133, 260 °C/5 kg)	EasyCork PLA	FormFutura	[60]
CL-PLA	PLA	Powdered stone / mineral powder	50/N.R.	1.7	HDT 55 °C, Tg 60 °C	MFR = 6 g/10 min (ASTM D1238, 210 °C/2.16 kg)	StoneFil	FormFutura	[61]
Cu-PLA	PLA	Dispersed metallic pigment particles	N.R.	1.24	Tg = 58-62 °C	N.R.	PLA silk copper	Wanhao	[62]
GF-PLA	Modified PLA	Glass fiber	16/N.R.	1.31	HDT 56 °C	MFI = 6.36 (GB/T 3682, 190 °C/2.16 kg)	PLA-GF	eSUN	[63]
Gr-PLA	PLA	Graphene flakes	5/N.R.	1.24	N.R.	MFR = 44.0 g/10 min (ISO 1133, 230 °C/2.16 kg)	PLA Graphene Strong Jet Black	Prografen	[64]
HF-PLA	Recycled PLA (rPLA)	Organic bio-fillers (hemp-related by-products)	N.R.	1.24	HDT 55 °C, Vicat 55-60 °C	MFR = 6 g/10 min (ASTM D1238, 210 °C/2.16 kg)	Organic rPLA - Hemp	FormFutura	[65]
LW-PLA	PLA	Foaming technology grade	N.R.	0.8 (at 23 °C)	Tg = 62.4 °C; Tm = 150.8 °C; Tc = 115.5 °C (DSC, 10 °C/min)	N.R.	PolyLite LW-PLA Grey	Polymaker	[66]
SP-PLA	PLA blend	Stainless steel powder	N.R./< 250	2.3	Molecular structure: Amorphous; Tm onset ≈ 155 °C	N.R.	Stainless Steel PLA	Proto-pasta	[67]
WF-PLA	PLA	Wood fibers / wood flour	N.R.	1.2	N.R.	MFI = 14 g/10 min (190 °C/2.16 kg)	Cardboard Spool-Wood	Elegoo	[68]

N.R. indicates 'not reported' in the manufacturer's publicly available datasheet/specification; values are listed here as provided by the corresponding source without extrapolation.

S2. Definition of derived properties and data processing

All dimensional, structural, and process-related descriptors were derived from the primary measurements according to consistent analytical definitions. Calculations were first performed at the specimen level and subsequently aggregated at the material level by computing mean values and standard deviations (SDs) across the four replicated specimens. No data filtering or outlier removal was applied at any stage of the analysis [30, 48-52].

♦ **Dimensional descriptors**

For each printed specimen, the signed relative dimensional deviation δ_i was calculated independently along the three measured directions: height (*h*) and the two orthogonal in-plane axes (b_1 and b_2). The relative deviation for a given axis *i* was defined as:

$$\delta_i(\%) = \frac{L_i - L_0}{L_0} \times 100 \quad (1)$$

where L_i represents the measured mean dimension along axis *i*, obtained by averaging the three local measurements on that axis, and $L_0 = 30\ mm$ is the nominal cube edge length.

To provide an axis-independent indicator of DA, the relative dimensional error *RDE* was defined as the absolute value of the relative deviation δ_i:

$$RDE_i(\%) = \langle |\delta_i| \rangle \quad (2)$$

A global volumetric dimensional deviation was computed to integrate the cumulative effect of deviations along all three axes. The measured specimen volume V_m was calculated as:

$$V_m = h \cdot b_1 \cdot b_2 \quad (3)$$

The volumetric deviation (ΔV) relative to the nominal cube volume $V_0 = 30^3\ mm^3$ was then expressed as:

$$\Delta V\% = \frac{V_i - V_0}{V_0} \times 100 \quad (4)$$

To quantify the out-of-plane vs in-plane dimensional anisotropy of the printed cubes, an anisotropy ratio AR was defined as the ratio between the absolute height deviation and the mean absolute in-plane lateral deviation:

$$AR = \frac{|\delta_h|}{(|\delta_{b1}| + |\delta_{b2}|)/2} \quad (5)$$

Here, δ_h, δ_{b1}, and δ_{b2} denote the signed relative deviations of the measured dimensions from the nominal cube dimensions along the height and the two in-plane directions, respectively; absolute values are used to compare deviation magnitudes.

♦ **Structural descriptors**

Bulk density ρ_{bulk} was determined from the measured specimen mass m_m and the geometrically determined measured volume V_m. Prior to density calculation, volumes expressed in mm^3 were converted to cm^3 to ensure dimensional consistency. ρ_{bulk} was therefore defined as:

$$\rho_{bulk} = \frac{m_m}{V_m} \quad (6)$$

Relative density *RD* was introduced as a normalized, dimensionless indicator of internal compactness, defined as the ratio between the measured bulk density ρ_{bulk} and the nominal material density ρ_{mat}:

$$RD = \frac{\rho_{bulk}}{\rho_{mat}} \quad (7)$$

Here, ρ_{mat} denotes the manufacturer-declared nominal density of the corresponding commercial filament grade (see Supplementary Table S1).

Based on RD, the internal porosity fraction ϕ was calculated as:

$$\phi(\%) = (1 - RD) \times 100 \quad (8)$$

To assess specimen-to-specimen repeatability in internal structure formation, the coefficient of variation of bulk density *CV* was calculated for each material as:

$$CV\% = \frac{\sigma_\rho}{\mu_\rho} \times 100 \quad (9)$$

where μ_ρ and σ_ρ are the mean and SD of ρ_{bulk} across the replicated specimens.

♦ **Process-related descriptors**

The mass deposition rate was calculated by normalizing the measured specimen mass m_m by the fixed MT of $t=76.75$ *min*.

The mass deposition rate R_m was defined as:

$$R_m = \frac{m_m}{t} \quad (10)$$

To account for material density effects on volumetric productivity, the build rate efficiency *BRE* was defined as:

$$BRE = \frac{R_m}{\rho_{mat}} \quad (11)$$

Extrusion stability was quantified through the mass flow irregularity index *MFII*, defined as the coefficient of variation of deposited mass across replicated specimens:

$$MFII = \frac{\sigma_m}{\mu_m} \quad (12)$$

where μ_m and σ_m denote the mean and SD of the measured specimen mass m_m.

A volumetric counterpart to MFII was introduced through the volumetric build stability index *VBSI*, calculated as:

$$VBSI = \frac{\sigma_V}{\mu_V} \quad (13)$$

with μ_V and σ_V represent the mean and SD of the measured specimen volume V_m.

The resulting datasets provide a consistent and statistically robust basis for comparative evaluation and multivariate analysis of the investigated materials.

In all density-derived calculations, the reference material density (ρ_{mat}) was taken as the manufacturer-declared nominal density of each commercial filament grade (Supplementary Table S1), while the bulk density of the printed specimens (ρ_{bulk}) was computed as mass-to-external-volume; relative density (RD) and porosity (ϕ) were then obtained from ρ_{bulk} and ρ_{mat} without reconstructing theoretical composite density from constituent-phase densities.
